# Morel-Lavallée lesion: A case report and literature review

**DOI:** 10.1097/MD.0000000000047083

**Published:** 2026-01-16

**Authors:** Yang Li, Wei Li, Lianrong Dou, Hongzhi Ding

**Affiliations:** a Department of Orthopaedic Surgery, Songjiang Hospital Affiliated to Shanghai Jiao Tong University School of Medicine, Shanghai, PR China.

**Keywords:** closed degloving injury, Morel-Lavallée lesion, sarcoma, soft tissue, trauma

## Abstract

**Rationale:**

Morel-Lavallée lesions (MLLs) represent closed degloving injuries resulting from traumatic separation of skin/subcutaneous tissue from deep fascia. While typically associated with high-energy trauma, diagnosis becomes challenging in cases with occult trauma, complex comorbidities, or tumor-mimicking imaging features. This case report aims to highlight the diagnostic challenges and management strategy of MLL in a patient with a history of malignancy and anticoagulant therapy, underscoring the importance of pathological confirmation in atypical presentations.

**Patient concerns:**

A 56-year-old woman was diagnosed with: MLL, hypertension, type II diabetes mellitus, status post meningioma resection, status post hysterectomy for cervical cancer, and status post inferior vena cava filter placement. Hip magnetic resonance imaging revealed soft tissue infiltration. Following comprehensive history review and pathological evaluation, malignancy was excluded, confirming the diagnosis of MLL.

**Diagnoses:**

Morel-Lavallée lesion.

**Interventions:**

Initial management included intravenous antibiotic therapy (cefazolin, 1 g twice daily) and percutaneous aspiration of fluid for pathological examination. Following exclusion of malignancy, the patient underwent debridement of peritrochanteric soft tissues with subsequent application of vacuum sealing drainage.

**Outcomes:**

The patient showed significant clinical improvement following percutaneous aspiration and antibiotic therapy. After surgical debridement and vacuum sealing drainage application, the wound healed completely without recurrence. She was discharged after an additional week of postoperative antibiotics and supportive care, with no complications at 1-month follow-up.

**Lessons:**

MLL require differential diagnosis from soft tissue neoplasms, particularly in patients with a history of malignancy. Anticoagulation may predispose to occult MLL development. Consequently, pathological examination is essential to establish a definitive diagnosis of MLL.

## 1. Introduction

The Morel-Lavallée lesion (MLL), a closed degloving soft tissue injury first delineated in 1853, occurs when skin and subcutaneous tissue abruptly separate from the underlying fascia, leading to the loss of vascular and lymphatic supply and subsequent formation of a cystic cavity containing blood, lymph, and necrotic fat.^[1,2]^ The lesions can develop in various anatomical locations, including the greater trochanter/hip (30.4%), thigh (20.1%), pelvis (18.6%), knee (15.7%), buttocks (6.4%), lumbosacral region (3.4%), abdomen (1.4%), lower leg/calf (1.5%), and head (0.5%).^[3]^ The presence of the MLL holds particular significance for orthopedic surgeons, as it may enhance the risk of perioperative infection from mixed fluid accumulation within the cystic cavity.^[4,5]^ Clinical manifestations of MLL vary with injury type and severity, ranging from localized pain (with or without restricted activity) to absence of symptoms. Signs of acute trauma include petechiae, soft tissue edema, fluctuations, and augmented skin mobility. Careful distinction between infections and tumors is imperative during clinical diagnosis, especially considering that tumors may emerge late in the lesion’s course, displaying subtler clinical indications than chronic injuries. Treatment modalities should be customized according to the lesion’s size, severity, and any accompanying injuries. These modalities include closed puncture aspiration, compression bandaging, local sclerotherapy, and incision and drainage. Chronic lesions or a patient’s history of tumors can mislead both the imaging team and the orthopedic surgeon, further contributing to misdiagnosis. The challenging diagnosis in this report’s patient, attributed to a history of tumors, oral anticoagulation treatment for thrombosis, ambiguous trauma history, and extensive cystic abnormalities, is discussed. Finally, the existing knowledge on MLLs is appraised, considering the specific characteristics of this case.

## 2. Case presentation

A 56-year-old woman presented to the oncology department of our hospital with symptoms of fatigue and joint discomfort that had persisted for 2 weeks. During the initial consultation, she sought orthopedic evaluation owing to pain and swelling localized to her right hip. Ultrasonography revealed a mixed mass in the lateral subcutaneous soft tissue of the upper right thigh. Magnetic resonance imaging (MRI) with contrast enhancement identified a soft tissue mass in the right lateral hip. This mass exhibited central liquefaction necrosis and marked enhancement of the solid portion, with involvement of the deep soft tissue. The attending radiologist suspected that the lesion might represent a soft tissue sarcoma, necessitating exclusion of a metastatic tumor.

Laboratory examination yielded the following results: white blood cell count at 10.01 × 10^9^/L (elevated); red blood cell count at 3.77 × 10^12^/L (decreased); and hemoglobin at 10^9^ g/L (decreased). Bacterial culture remained negative. According to the patient’s recent history, she had sustained a slight abrasion to the right hip approximately 2 weeks prior, accompanied by mild localized pain and preserved mobility of the affected limb. Past medical history included: 11 years of hypertension managed with irbesartan hydrochlorothiazide (150 mg, qd, Po); 20 years of diabetes, currently treated with Novolin R, 20U subcutaneously injected before breakfast and dinner; a hysterectomy performed 4 years prior for cervical cancer; and meningioma removal 19 months ago. Additionally, 2.5 months ago, the patient underwent “lower extremity venography + lower extremity venous filter implantation” due to deep vein thrombosis, with subsequent oral administration of rivaroxaban (10 mg, qd, Po).

**Figure 1.**
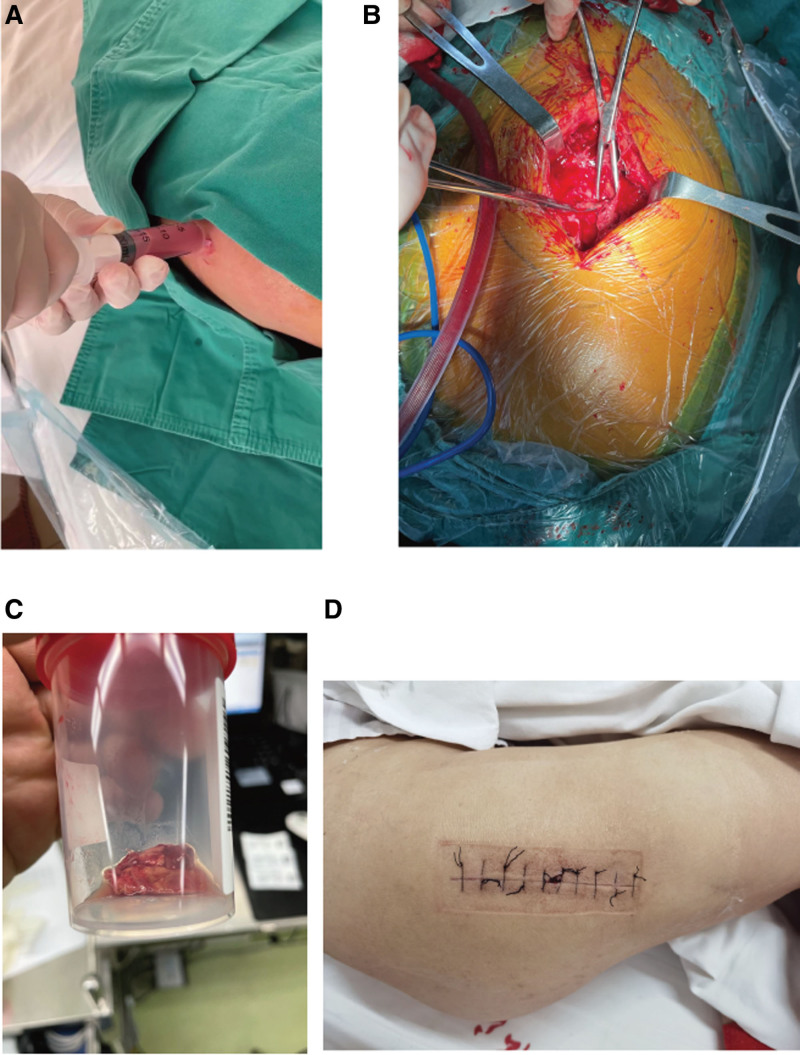
(A) Percutaneous puncture test, with puncture fluid earmarked for pathological biopsy and bacterial culture. (B) Surgical treatment, detailing intraoperative debridement of the lesion and deeper exploration for comprehensive lesion clearance. (C) Intraoperative conservation of a portion of the tissue to undertake a subsequent pathological biopsy, intended to ascertain the specific nature of the lesion. (D) Postoperative positioning of a negative pressure suction device, enabling cavity closure and fostering favorable wound healing.

**Figure 2.**
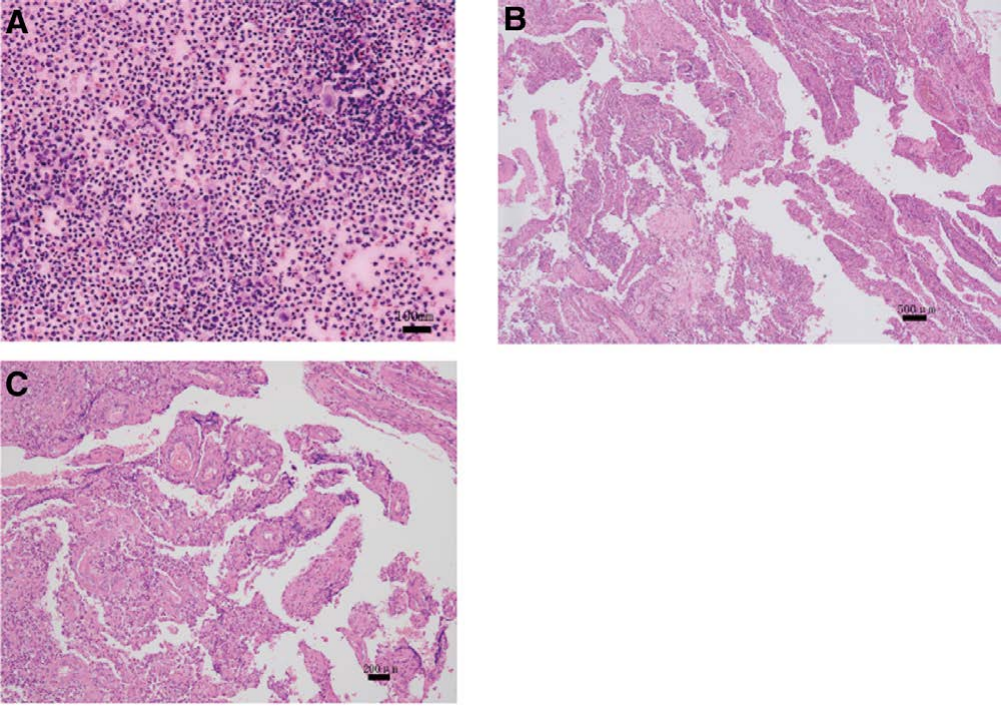
(A) Pathological findings from percutaneous aspiration fluid, revealing abundant neutrophils and cells with enlarged nuclei, with no evidence of malignant cells. (B, C) Histopathological examination of tissue specimens demonstrates inflammatory granulation tissue featuring capillary hyperplasia and dense infiltration by lymphocytes and neutrophils (B) magnification 40×; (C) magnification 100×.

Initial treatment included intravenous antibiotics (cefazolin, 1 g twice daily) and aspiration of 80 mL of slightly bloody, turbid fluid for pathological examination(Fig. 1A). Pathological examination of the aspirate revealed numerous neutrophils and macronucleus, with no evidence of malignant cells (Fig. 2A). Correlating these findings, the preliminary diagnoses were established as: MLL; hypertension; type II diabetes; post-meningioma surgery; post-hysterectomy for cervical cancer; and post-implantation of inferior vena cava filter.

Further therapeutic measures included soft tissue debridement and vacuum sealing drainage (VSD) around the hip, with retention of the excised soft tissue for subsequent pathological evaluation (Fig. 1). Microscopic examination revealed inflammatory granulation tissue, capillary hyperplasia, and an abundant presence of lymphocytes, histiocytes, and neutrophils, along with abscess formation (Fig. 2B, C). The possibility of sarcoma and metastasis was definitively excluded through pathological biopsy, confirming the diagnosis of MLL with concomitant infection. Following a single closed aspiration and a 2-week course of antibiotics, the patient’s symptoms alleviated. Further improvement was observed after debridement and VSD suction (6 days in total, resulting in 151 mL of drainage fluid), leading to wound healing. The patient was discharged after an additional week of postoperative antibiotic therapy and supportive care. At the 1-month follow-up, the wound had healed completely, with no signs of recurrence or infection, and the patient reported full functional recovery.

## 3. Discussion

### 3.1. Epidemiology

MLLs often arise from the relative displacement of the skin and subcutaneous tissue away from the deep fascia as a result of car accidents or sudden shearing forces. Motor vehicle collisions are the predominant cause, with high-energy mechanisms accounting for 25%.^[6,7]^ Additionally, MLLs have been associated with iatrogenic injuries observed during long-term follow-up post breast surgery and abdominal liposuction,^[8,9]^ as well as sports injuries and minor violent mechanisms.^[10-12]^ The epidemiological attributes of MLL contribute to challenges in clinical diagnosis. Comprehensive collection of detailed history is paramount, especially when individuals have endured mild and difficult-to-recall traumatic experiences. In the present case, detailed questioning regarding the medical history facilitated the patient’s recall of minor trauma. When considered in conjunction with her history of anticoagulant usage, MLL was suspected as the underlying etiology.

### 3.2. Pathological and anatomical features

**Figure 3.**
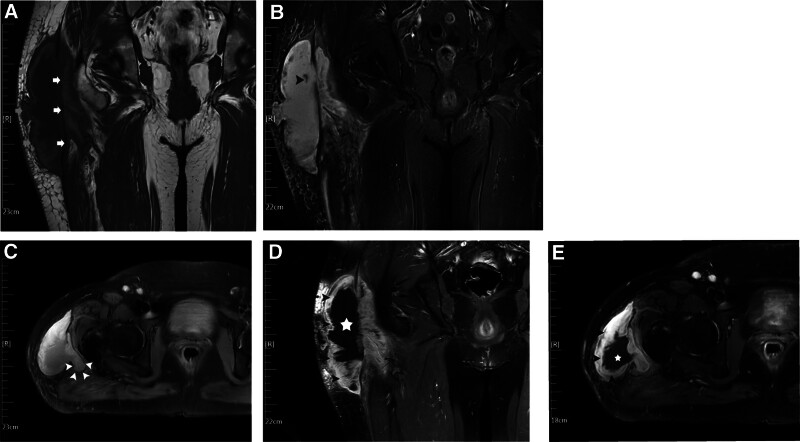
(A) T1-weighted coronal image presents an irregular soft tissue mass with mixed signals between the adipose layer and the deep fascia of the right hip, demonstrating an isohigh signal, partially engaging the deep tissue, and spanning approximately 185 mm in length; gray arrows: adipose layer; white arrowheads: deep fascia. (B) STIR sequence reveals adipose tissue signal suppression, while certain regions within the lesion exhibit a low signal (short gray arrow), indicative of fat particles. (C) STIR in the transverse section elucidates that the lesion penetrates the deep fascia and extends into the deeper tissues; short white arrows: the lesion penetrating the deep fascia. (D, E) Coronal and cross-sectional enhancement images display perilesional enhancement with a low signal centrally and encroachment of the lesion into both the fat layer and the deeper strata of the deep fascia; short black arrows: enhancement of the perilesional tissue; white pentagrams: low-signal central region of the lesion. STIR = short tau inversion recovery.

Trauma can cause detachment of the skin and subcutaneous adipose tissue from the underlying fascia, resulting in shearing damage to capillaries and lymphatics, with consequent accumulation of lymph, blood, debris, and fat forming a potential cavity. This fluid accretion with mixed components may progress gradually or rapidly. Eventually, the blood is resorbed and substituted with serous fluid.^[13]^ Persistent effusion can result in the formation of a large mass, often misdiagnosed as a tumor. Inflammatory response culminates in the final pathological transformation, forming a fibrous capsule with a cyst-like structure; thus, MLL is also termed a post-traumatic pseudocyst. Such a structure may facilitate MLL recurrence and slow growth through exudation.^[14]^ Nevertheless, reports indicate that lesions may diminish in size over time and self-resolve.^[15]^ In this case, considering the patient’s prior tumor history, the radiologist initially suspected a tumor or metastasis based on MRI findings. Therefore, we advocate that post-puncture pathological examinations, including immunohistochemical tests when necessary, supplement conventional noninvasive modalities (computed tomography [CT], ultrasound, and MRI) to provide more conclusive evidence for definitive diagnosis and tumor differentiation. Concurrently, we observed that the pathological alterations in this patient were not as localized as classic MLL, with MRI-visible invasion into deep tissue (Fig. 3).

### 3.3. Clinical manifestations

Typical clinical presentations of MLL include a painful, enlarging lesion subsequent to local trauma, commonly detected within hours to days, characterized by local pain, swelling, and stiffness. However, up to a third of patients may only be diagnosed months or even years post-trauma.^[1]^ MLL may present solitarily but is often concurrent with fractures and may occur unilaterally or bilaterally.^[2,16]^ MLLs are commonly located in bony prominences, particularly the trochanteric region and proximal femur,^[17]^ as well as other regions like the knee, calf, and abdomen. Localized skin frequently exhibits reduced sensation due to subcutaneous nerve damage.^[18]^ Physical examination typically reveals soft, fluctuant areas of the overlying skin.^[1,13]^ Some patients may develop skin necrosis due to direct injury or ischemia from expansion of the subcutaneous space.^[1,19]^ Although MLL is a closed injury, associations with bacterial infection have been reported, and bacterial growth can yield positive outcomes.^[16,17,20,21]^ In this case, the clinical manifestations were subtle, with sudden slight swelling and pain surrounding the large hip nodules, no discernible skin surface abnormality, and no evidence of fluctuation. Consequently, we emphasize that patients suspected of MLL require meticulous physical examination, complemented by targeted imaging examination, founded on the respective physical findings.

### 3.4. Ultrasound, CT, and MRI as auxiliary examinations

**Figure 4.**
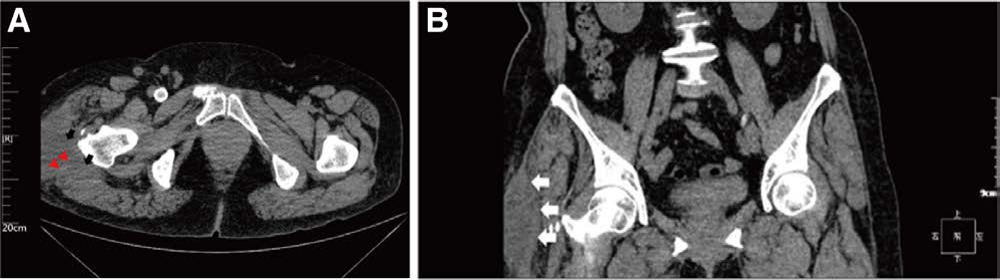
(A) Computed tomography (CT) plain scan of the patient’s pelvis in the transverse position illustrates a slightly hypodense soft tissue shadow within the lateral and posterior subcutaneous tissue of the left hip, as indicated by the black arrows. Apparent separation within the region is depicted by the red arrowheads. (B) On coronal non-contrast CT imaging of the pelvis, an elliptical, slightly hypodense soft tissue lesion is visualized within the subcutaneous tissue lateral to the left hip (indicated by the white arrow). CT = computed tomography.

In the clinical diagnosis of MLLs, ultrasound, CT, and MRI are regularly employed. Thus, it is essential for clinicians and radiologists to comprehend both acute and chronic manifestations of MLLs to mitigate the risk of incorrect or missed diagnoses. Often, clinicians initiate the examination process by assessing x-ray films for fractures. MLLs frequently manifest as local, nonspecific soft tissue swelling on plain films, and may be concurrent with fractures.^[22]^ Due to the precipitation of blood components and variations in blood and lymph contents, MLLs often display a fluid-fluid plane, with a density usually lower than that of simple hematomas as a result of the presence of low-density lymph.^[23,24]^ Lesions less than a month old tend to have irregular margins, whereas chronic lesions, which are more prevalent, exhibit uniform densities, regular margins, and often encapsulation.^[25]^ In the CT scan of this patient’s pelvis, a slightly low-density soft tissue shadow on the left hip and a lesion spanning the deep fascia are evident (Fig. 4).

### 3.5. Ultrasound imaging

**Figure 5.**
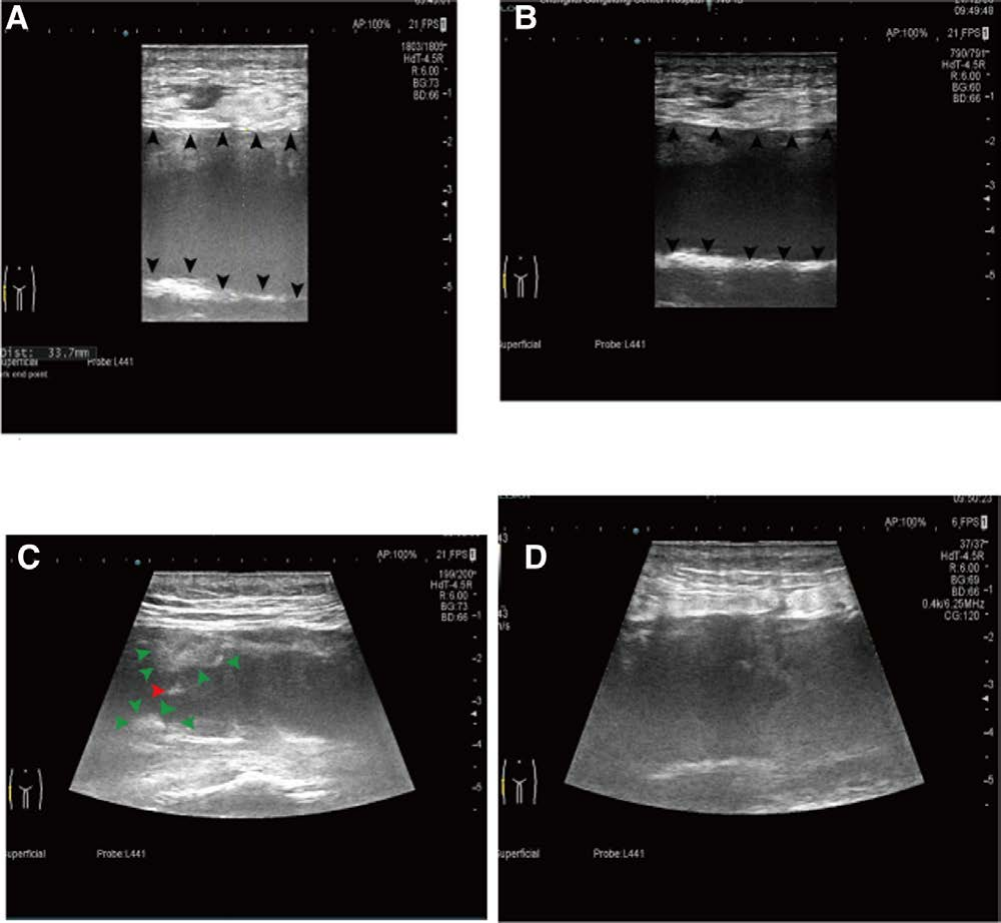
Ultrasound imaging of the right proximal femur reveals an inhomogeneous collection of mixed echogenicity. (A, B) The mixed echogenic collection is shown beneath the fat layer, with arrows demarcating its longitudinal upper and lower boundaries. (C) An irregular protuberance on the inner surface of the mixed collection is represented in green, while a potentially highly echogenic fat particle is denoted in red. Color Doppler flow imaging. (D) CDFI did not manifest any discernible blood flow signal. CDFI = color Doppler flow imaging.

The appearance of MLLs on ultrasound is intricately related to lesion duration and the evolution of blood components. Acute and subacute lesions (under 1 month) present heterogeneity with irregular, lobular margins. In contrast, chronic lesions (exceeding 18 months) are more uniform, displaying smooth, flattened or spindle-shaped margins.^[26]^ On color Doppler imaging without blood flow, all lesions were found to be compressible,^[27]^ and fat might manifest as hyperechoic nodules.^[23]^ Figure 5 illustrates an ultrasound examination of this patient, revealing a lesion measuring 185 mm × 34 mm × 83 mm, characterized by a defined border, slightly irregular shape, uneven internal echo, and a combination of cystic and solid features. The color Doppler flow imaging detected no discernible blood flow signal.

### 3.6. MRI imaging

MRI is the preferred imaging method for diagnosing MLL injuries; however, its manifestations are intricately related to the characteristics of hemorrhage and blood degradation products. The imaging characteristics of MLLs on MRI typically include a fusiform fluid collection between the subcutaneous fat and fascia, with variations in signal intensity on T1- and T2-weighted images depending on the lesion’s content and chronicity.^[27,28]^ MRI’s ability to classify MLLs into different types, such as seroma, subacute hematoma, and chronic organizing hematoma, aids in tailoring the management approach to the specific characteristics of the lesion.^[29]^ This classification is critical, as it informs the decision-making process regarding the most appropriate treatment strategy, whether it be conservative management, percutaneous drainage, or surgical intervention.^[30]^ Figure 3 depicts an irregular, large soft tissue mass located beneath the right hip’s lateral fat layer, characterized by mixed signals, isohigh signal in T1, and high signal in Stir. Notably, the peripheral part of the lesion demonstrated significant enhancement after the application of contrast, while the central portion did not. The lesion also extended into deeper soft tissues. Although the MRI findings were consistent with the patient’s several weeks of ailment, the involvement of the lesion in deeper tissues both complicated the diagnosis and increased the potential for misdiagnosis.

### 3.7. Differential diagnosis

MLLs demand an extensive range of differential diagnoses, especially when they manifest as chronic lesions of indeterminate origin. These primarily encompass differentiation from bursitis and sarcoma. When a lesion progressively enlarges or elicits pain without an evident history of trauma, there is potential for MLLs to be misconstrued as a superficial soft tissue tumor. MRI is particularly instrumental in the differential diagnosis between MLLs and soft tissue tumors. Chronic MLLs may be discerned if they exhibit an oval or spindle-shaped morphology with an oval margin, are situated posterior to subfascial fat dissection, and are positioned in a typical location adjacent to the fascia lata within the perifascial plane.^[13]^ A distinguishing characteristic of MLLs as compared to sarcomas is a pronounced, uniform enhancement of internal contrast, a feature not observed in sarcomas.^[31]^ Biopsy can occasionally aid in confirming a diagnosis.

In the context of the knee’s anterior region, MLLs must be differentiated from prepatellar bursitis. Notably, the knee’s prepatellar region is a prevalent site for bursitis, a condition that predominantly afflicts individuals engaged in professions like maids, wrestlers, and other occupations necessitating similar motions.^[32]^ Further, given the analogous clinical features between periknee MLLs and prepatellar bursitis, MRI may be warranted to differentiate these 2 entities.^[33]^ Unlike MLLs, prepatellar bursitis is more localized and seldom extends beyond the coronal midline and mid-femur.^[10]^ Relying solely on size range for differentiation^[32,33]^ may not yield precise results. In instances where size does not provide clear distinction, subsequent imaging and the lesion’s response to steroid injection may be employed for diagnosis. Most cases of prepatellar bursitis naturally resolve as blood products are reabsorbed or excreted, leaving no residual damage, whereas alterations in MLLs are typically negligible. Moreover, the degree of change in MLLs in response to steroid injection is less conspicuous than that observed in bursitis.^[33]^

In the present case, the preliminary diagnosis was oriented towards soft tissue sarcomas surrounding the hip. However, a review of the patient’s history uncovered previous hip trauma. When considered in conjunction with the specific location around the greater femoral trochanter, this led to a reevaluation of the initial diagnosis. The biopsy findings, including the presence of inflammatory cells and megakaryocytes but an absence of tumor cells, further substantiated the diagnosis of MLLs. Ultimately, the final postoperative pathological biopsy results affirmed the diagnosis of MLLs.

### 3.8. Treatment

MLLs present various therapeutic avenues, bifurcated into non-surgical and surgical modalities. The non-surgical spectrum encompasses rest, compression, physiotherapy, and local aspiration, while the surgical paradigm includes options such as open or percutaneous drainage, with or without sclerotherapy. Existing literature has posited an algorithm for treatment predicated on lesion acuity.^[34]^ For acute lesions with closed or absent underlying fracture, initial intervention with compression bands, with or without concomitant sclerotherapy, is recommended, followed by percutaneous drainage for lesions unresponsive to compression bands. If percutaneous drainage does not achieve resolution, or if the lesion is concomitant with an open underlying fracture, open debridement becomes necessary. Chronic lesions, conversely, should be initially approached with percutaneous drainage and sclerotherapy. It merits noting that the absence of sclerotherapy in the treatment of chronic lesions may precipitate postoperative hematoma recurrence and secondary infection.^[26]^ Sclerotherapy has emerged as a promising adjunctive treatment for MLLs, particularly in cases where conservative management has failed or when the lesion is recurrent. Sclerotherapy involves the injection of a sclerosing agent, such as doxycycline, into the lesion to promote fibrosis and obliteration of the fluid collection. This technique has been shown to be effective in reducing recurrence rates and improving patient outcomes when used in conjunction with ultrasound-guided aspiration.^[35,36]^ The combination of MRI for diagnostic precision and sclerotherapy for treatment highlights a multimodal approach to managing MLLs, which can be particularly beneficial in atypical or refractory cases.^[35]^ As the understanding of MLLs continues to evolve, further research into standardized treatment protocols and long-term outcomes is warranted to optimize patient care.^[35,37]^ Persistent lesions may call for surgical debridement, 1-stage closure, regular compression, and sclerotherapy. Lesions that are recalcitrant to treatment or become infected may require open drainage and secondary closure. Perineal tear lesions, associated with significant morbidity and mortality, may necessitate iterative debridement, vacuum closure drainage, skin grafts, genital reconstruction, and anoplasty.^[38]^ In the present case, owing to the encapsulation of the lesion, open debridement was executed post puncture aspiration and pathological biopsy to substantiate the diagnosis and exclude malignancy. Subsequent surgery employed a VSD device for continuous negative pressure suction to excise the lesion and secure a favorable outcome (as depicted in Fig. 1).

## 4. Conclusion

This manuscript delineates a systematic review of MLLs, focusing on a case example. Diagnostic challenges pervade the terrain of MLLs, mandating unequivocal diagnosis within the continuum of patient care. Acute MLLs are particularly enigmatic in diagnosis. Chronic manifestations, marked by delayed clinical appearance, often render it arduous for patients to recount explicit trauma history, and the heterogeneity in imaging findings of chronic lesions necessitates differentiation from other soft tissue lesions or even soft tissue tumors, thus elevating the propensity for misdiagnosis. In this instance, the patient’s prior tumor history and intricate medical past further obscured definitive diagnosis. Understanding the pathological and anatomical characteristics of MLLs is paramount; the separation of skin and subcutaneous adipose tissue from the underlying fascia due to shear force, and the rupture of blood and lymphatic vessels culminating in localized fluid accumulation, typifies MLLs. Common in areas rich in subcutaneous fat like hips and thighs, MLLs were once considered confined to deep fascia and subcutaneous tissue. However, this case has provided new insights, with co-infection and pressure release into deep tissue being plausible culprits for this anomalous manifestation. For initial imaging diagnosis, MRI emerges as the preferred modality, and may be augmented with traditional imaging techniques such as ultrasound and CT for auxiliary diagnosis. Enhanced MRI is particularly vital for differentiating MLLs from soft tissue tumors. MRI findings for chronic lesions may be variable, and in cases where definitive diagnosis remains elusive, a tissue biopsy is advisable. In conclusion, most MLLs are amenable to treatment, primarily with sclerotherapy or negative pressure therapy to remove the lesion, close the cavity, and promote healing.

## 5. Limitations

This study has several limitations. First, it is a single-case report, which limits the generalizability of the findings. Second, the retrospective nature of the study and the delayed ethical approval are acknowledged as procedural shortcomings. Third, the patient’s complex medical history and the absence of initial trauma history posed diagnostic challenges that may not be universally applicable. Future prospective studies with larger cohorts are needed to validate our findings.

## Acknowledgments

The authors thank the patient for providing written informed consent for the publication of this case report and accompanying images.

## Author contributions

**Conceptualization:** HongZhi Ding.

**Data curation:** Yang Li.

**Formal analysis:** Yang Li, Wei Li, Lianrong Dou, HongZhi Ding.

**Resources:** HongZhi Ding.

**Software:** Yang Li, HongZhi Ding.

**Writing** – **original draft:** Wei Li, HongZhi Ding.

**Writing** – **review & editing:** Lianrong Dou, HongZhi Ding.
